# Complete Genome Sequence of Desulfovibrio desulfuricans IC1, a Sulfonate-Respiring Anaerobe

**DOI:** 10.1128/MRA.00456-19

**Published:** 2019-08-01

**Authors:** Leslie A. Day, Kara B. De León, Megan L. Kempher, Jizhong Zhou, Judy D. Wall

**Affiliations:** a Department of Biochemistry, University of Missouri, Columbia, Missouri, USA; b Institute for Environmental Genomics, Department of Microbiology and Plant Biology, University of Oklahoma, Norman, Oklahoma, USA; University of Southern California

## Abstract

We report the complete genome sequence of the anaerobic, sulfonate-respiring, sulfate-reducing bacterium Desulfovibrio desulfuricans IC1. The genome was assembled into a single 3.25-Mb circular chromosome with 2,680 protein-coding genes identified. Sequencing of sulfonate-metabolizing anaerobes is key for understanding sulfonate degradation and its role in the sulfur cycle.

## ANNOUNCEMENT

The anaerobic sulfate-reducing bacterium (SRB) Desulfovibrio desulfuricans IC1 has been shown to utilize selected sulfonates, compounds that contain an R-C-SO_3^−^_ moiety in respiration (1). Sulfonates are ubiquitous in nature, are synthesized commercially (e.g., detergents), and may be serving as key metabolites for SRB, in sulfate-limited environments.

The genes involved in sulfonate degradation by aerobic bacteria have been extensively investigated (2). However, exploration of the genes and biochemistry of sulfonate respiration by anaerobes is incomplete. Therefore, the sequencing and analysis of sulfonate-utilizing anaerobes may reveal novel metabolic pathways for the metabolism of organosulfur compounds.

*D. desulfuricans* IC1 was isolated with the sulfonate isethionate as the terminal electron acceptor from freshwater marsh mud (1). We acquired *D. desulfuricans* IC1 from Leibniz Institute DSMZ (DSM no. 12129). For sequencing, *D. desulfuricans* IC1 was grown at 30°C in MOYLS4 medium (3). The genomic DNA (gDNA) was extracted with a Wizard genomic DNA purification kit (Promega Corp., Madison, WI, USA) per the manufacturer’s guidelines for Gram-negative bacteria, and the DNA was resuspended in Tris-HCl (10 mM, pH 8.5). The DNA was quantified with a Qubit fluorometer and Qubit double-stranded DNA (dsDNA) broad-range (BR) assay kit (Thermo Fisher Scientific, Waltham, MA, USA).

Sample preparation and MiSeq (Illumina, San Diego, CA, USA) sequencing of *D. desulfuricans* IC1 was performed by the University of Oklahoma Institute for Environmental Genomics as follows: 1 μg of gDNA was fragmented with a Covaris M220 focused ultrasonicator, and the library was prepared with the Kapa HyperPrep kit (Kapa Biosystems) following the manufacturer’s protocol. Paired-end sequencing was performed with the MiSeq v2 500-cycle reagent kit (Illumina). All software used in this study was operated with default parameters. ϕX174 (GenBank accession no. NC_001422.1) reads and adaptor sequences were removed with Bowtie 2 (4) and Cutadapt (v1.18) (5), respectively, resulting in 4,294,900 paired-end reads with an average length of 163 nucleotides.

A 20-kb SMRTbell long-insert size-selected library was prepared and analyzed on the PacBio (Menlo Park, CA, USA) RS II platform with single-molecule real-time (SMRT) sequencing technology at the University of Maryland Institute for Genome Sciences. Sequencing produced 156,863 raw reads with an average length of 8,203 nucleotides. Reads were trimmed, corrected, and assembled with Canu (v1.7) into one circular contig with an average coverage of 39.1 (6). Trimmed Illumina reads were used to correct insertions and deletions in the PacBio assembly with Pilon (v1.21) (7).

Illumina sequencing reads were mapped to the PacBio assembly in Geneious (v9.0.5; Biomatters, Ltd., Auckland, New Zealand), with an average coverage of 180.2. The chromosome starting position was designated as 150 bases upstream of *dnaA.* One single-nucleotide polymorphism (SNP) was identified in the population with the Geneious find variations/SNPs function. This SNP is at position 1638748 in the gene for a TetR/AcrR family transcriptional regulator (DDIC_06845) with a 50/50 occurrence of G/T. The BLAST function in Geneious revealed a gene encoding a putative glycyl radical enzyme (DDIC_12990) in *D. desulfuricans* IC1 which has 77% amino acid identity to the Bilophila wadsworthia protein (GenBank accession no. EFV45544.1) identified in sulfonate respiration (8).

The NCBI Prokaryotic Genome Annotation Pipeline identified 2,680 protein-coding genes, 1 clustered regularly interspaced short palindromic repeat (CRISPR) array, 3 rRNA operons, 54 tRNA, and 42 pseudo genes (9). The G+C content is 59.1% with a total length of 3,251,440 nucleotides.

### Data availability.

This whole-genome shotgun project has been deposited in DDBJ/ENA/GenBank under accession no. CP036295. This is the first version (CP036295.1). Raw PacBio sequences were deposited in the NCBI SRA database under accession no. SRR8582205. Raw Illumina sequences were deposited under SRA accession no. SRR7655955.
